# P07 Use of the Reveal rapid antimicrobial susceptibility testing system to shorten time to appropriate antibiotic use: assessment of clinical utility in Sheffield Teaching Hospitals NHS Trust

**DOI:** 10.1093/jacamr/dlad066.011

**Published:** 2023-06-14

**Authors:** Emma Williams, Joanne Fowler, Mary Gray, Mark Tovey, Nicole Rayworth, Lisa Tilley, Helena Parsons

**Affiliations:** Sheffield Teaching Hospitals NHS Trust, Sheffield, UK; Sheffield Teaching Hospitals NHS Trust, Sheffield, UK; Sheffield Teaching Hospitals NHS Trust, Sheffield, UK; Sheffield Teaching Hospitals NHS Trust, Sheffield, UK; Sheffield Teaching Hospitals NHS Trust, Sheffield, UK; Sheffield Teaching Hospitals NHS Trust, Sheffield, UK; Sheffield Teaching Hospitals NHS Trust, Sheffield, UK

## Abstract

**Objectives:**

Gram-negative bacteraemia accounts for significant morbidity and mortality,^1^ while increasing the prevalence of resistant organisms over the recent years^2^ highlights the importance of obtaining rapid antimicrobial susceptibility testing (AST) results. Antimicrobial stewardship (AMS) is part of the WHO global strategy on antimicrobial resistance,^3^ and shortening the length of time a patient is treated with unnecessarily broad-spectrum agents is paramount. Our aim was to review whether the Reveal rapid AST system (bioMérieux) shortened time-to-best-fit antibiotic, and whether this was a spectrum escalation or de-escalation, in Gram-negative bacteraemias in Sheffield Teaching Hospitals NHS Trust.

**Methods:**

The Reveal rapid AST system was used, in conjunction with standard laboratory methodology, to ascertain antimicrobial susceptibilities for Gram-negative bacilli in blood cultures processed at the start of the day between June and August 2022. Data was gathered regarding Medical Microbiology advice issued to clinical teams and the impact the Reveal AST results had on this. For comparison, we retrospectively reviewed microbiology advice given for Gram-negative bacteraemias between April and May 2022, with our routine microbiology testing methods of MALDI-TOF MS and breakpoint agar dilutions AST, to see whether Reveal would have been predicted to have an impact had it been available.

**Results:**

66 bacteraemias were reviewed between June and August 2022, compared with 82 in April and May 2022. Reveal results shortened time-to-best-fit antibiotic in 35/66 (53%) cases. Of these, 8/66 (12%) resulted in antibiotic spectrum escalation and 21/66 (32%) in de-escalation, with 6/66 (9%) resulting in shorter time to an alternative susceptible antibiotic of similar spectrum. In the retrospective cohort, earlier susceptibility results would have been predicted to shorten time-to-best-fit antibiotic in 53/82 (65%) cases [escalation 16/82 (20%), de-escalation 37/82 (45%)], consistent with the study period findings. Extrapolating this to cultures received throughout 2022 in our trust (*n* = 1722), with a mean AST turnaround time of 491 min compared with 1553 min for standard AST testing, the Reveal system could save 4877 hours in time to escalation and 11 887 hours in time to de-escalation to appropriate antibiotic.

**Conclusions:**

In our experience, Reveal resulted in a shortening of time-to-best-fit antibiotic in the majority of patients with Gram-negative bacteraemia. The impact on escalation may impact on mortality and morbidity, while de-escalation could have significant benefit in terms of AMS, with possible implications for shortened time to oral switch and shortened length of stay, with associated financial, operational and quality of care benefits.
